# 20 abnormal metabolites of Stage IV Grade C periodontitis was discovered by CPSI-MS

**DOI:** 10.3389/pore.2022.1610739

**Published:** 2022-12-07

**Authors:** Li-Jun Wang, Liu Liu, Wei Ju, Wen-Xin Yao, Xi-Hu Yang, Wen-Hao Qian

**Affiliations:** ^1^ Department of Periodontitis, Xuhui District Dental Center, Shanghai, China; ^2^ Department of Oral and Maxillofacial-Head & Neck Oncology, Ninth People's Hospital, School of Medicine, Shanghai Jiao Tong University, Shanghai, China; ^3^ Department of Oral and Maxillofacial Surgery, Affiliated Hospital of Jiangsu University, Zhenjiang, Jiangsu, China

**Keywords:** metabolomics, point-of-care test, conducting polymer spray ionization, saliva metabolomics, severe periodontitis (Stage IV and Grade C)

## Abstract

Saliva is a noninvasive biofluid that contains the metabolic signature of severe periodontitis (SP, Stage IV and Grade C). Conductive polymer spray ionization mass spectrometry (CPSI-MS) was used to record a wide range of metabolites within a few seconds, making this technique a promising point-of-care method for the early detection of SP (Stage IV and Grade C). Saliva samples from 31 volunteers, consisting of 16 healthy controls (HC) and 15 patients with SP (Stage IV and Grade C), were collected to identify dysregulated metabolites. Twenty metabolites were screened out, including seven amino acids. Moreover, the results showed that amino acid metabolism is closely related to the development of periodontitis. The present study further confirmed that salivary metabolites in the oral cavity were significantly altered after plaque removal. These results suggest that the combination of CPSI-MS is a feasible tool for preclinical screening of SP (Stage IV and Grade C).

## Introduction

Periodontitis is a chronic inflammatory disease, if left untreated, that results in loss of the natural dentition and absorption of alveolar bone. It is common in adults and has a high incidence worldwide. The current understanding about the etiology of periodontitis is that this disease is caused by dysbiosis between the host response and the bacterial aggression that colonizes dental surfaces. At present, the diagnosis of periodontitis mostly depends on clinical and radiographic examinations, and then mechanical root debridement has been taken, while the specific pathogenesis of periodontitis has not yet been clarified [1].

Recently, medical fields have begun to use omics technology to add knowledge to the biomarkers of periodontitis [2–4]. Metabolomics is a branch discipline of omics that focuses on changes in metabolites and metabolic pathways to understand the pathophysiological processes in biological systems. A variety of samples are used for metabonomics studies, including saliva, blood, urine, or tissue. Saliva is a viable biofluid of oral diseases that contains many of the same biomarkers as blood and serum [5]. It is composed of a variety of locally synthesized and systemically derived molecules involved in various metabolic processes and is influenced by interactions that occur between the patient, microbiome, and environmental factors [6, 7]. Importantly, the saliva samples of a patient can be collected easily and non-invasively and have revealed specific metabolomic signatures for numerous oral and systemic diseases [8].

Consequently, metabolomics can give valuable information in different phases of periodontal diseases using spectroscopic assay techniques[9]. Nuclear magnetic resonance (NMR) and mass spectrometry (MS) were used for high-throughput identification and quantification of metabolites in biological fluids [10–12]. Several studies have identified that the metabolome of saliva samples collected from chronic periodontitis patients is different from that of healthy controls, reaching discrimination accuracies of up to 84% [13,14].

Gas chromatography mass spectrometry (GC-MS), liquid chromatography mass spectrometry (LC-MS), and nuclear magnetic resonance spectrometry (NMR) are the most widely used metabolomic methods. In recent years, ambient ionization mass spectrometry has gradually gained interest in the field of clinical diagnosis because it is free from laborious pretreatment, having a wide coverage of metabolites, with high-throughput metabolome information monitoring among various biological samples. Combined with machine learning techniques for high-dimension data interpretation, it can be performed with comparable accuracy at a lower cost [15–18].

Our previous studies have reported the practical value of conductive polymer spray ionization mass spectrometry combined with machine learning (CPSI-MS/ML) for the discrimination of oral squamous cell carcinoma (OSCC) from premalignant lesions (PML) and healthy contrast (HC) [19]. CPSI-MS/ML has shown its advantage in directly collecting hundreds of metabolite abundant data from trace dried biofluid spots within a few seconds under atmospheric conditions [20]. The characteristic metabolites previously discovered in saliva were mainly narrowed to small molecules with molecular weights of less than 500 da.

The present study aimed to determine the salivary metabolic profile of patients with chronic severe periodontitis (SP Stage IV and Grade C) using CPSI-MS, thereby providing an alternative method for SP diagnosis.

## Methods

### Specimens collection and preparation

All study experiments were conducted in compliance with the ethical guidelines established by the Xuhui District Dental Center. Periodontal examination was performed at Xuhui District Dental Center by professional dentist who were previously trained and calibrated for the evaluation and sampling procedures. PPD, bleeding on probing, and CALwere recorded at 6 sites per tooth. According to the 2018 classification, Stage IV and Grade C periodontitis was defined as the most severe site with CAL≥5 mm, alveolar bone resorption up to 1/2-2/3 of the length of the root, the number of teeth lost due to periodontitis is greater than or equal to five and CAL loss greater than or equal to 2 mm within 5 years. The healthy control group was those without probe bleeding, loss of attachment and bone loss. A total of 46 saliva samples were collected from 31 volunteers, including 16 HCs and 15 Stage IV and Grade C periodontitis patients (pre- and post-treatment), and all participants signed informed consent forms. Saliva was collected from 15 patients with severe periodontitis 15 min after nonsurgical periodontal therapy (NST). Saliva was prepared by centrifugation at 3,000 rpm for 5 min at room temperature. All saliva samples were stored at −80°C until use. All experimental protocols were approved by Xuhui District Dental Center.

### Metabolomic profiling by CPSI-MS

After the saliva was thawed under ambient conditions, 3 μl saliva and 1 μl 4-chloro-phenylanine (internal standard) were transferred onto the tip of a conductive polymer to form a dried saliva spot for data acquisition. Upon the addition of methanol water (7:3, v/v, 3 μl) to the dried saliva spot for metabolite extraction, a +4.5 kV high voltage was applied to the conductive polymer tip to trigger the spray ionization process and data recording using an LTQ Orbitrap Velos mass spectrometer (Thermo Scientific, San Jose, CA, United States). . The full scan range was set at *m/z* 50-1000 in positive mode. The data acquisition period for each case lasted 15 s to collect sufficient metabolomic data. The intensity of each metabolite ion was normalized to the average total ion current (TIC) or IS the intensity (*m/z* 222.0298 [M + Na]^+^) of each sample. Quality control (QC) samples were prepared by pooling equal volumes of 20 NC and 20 OSCC saliva samples. QC samples were analyzed throughout the run to monitor variations in the CPSI-MS system. OSCC and NC sera were alternatively arranged for the test run with the QC samples evenly inserted into the entire sequence for every 30 samples. Refer to our previous reports for specific methods and steps [19, 20].

### Metabolomics data processing

All raw files were first converted into cdf format using Xcalibur (Thermo Fisher Scientific, San Jose, CA, United States) and then imported into MATLAB 2020a (MathWorks, Natick, MA, United States) for batch data preprocessing using a self-programmed script. The metabolomic profile of each sample were obtained by averaging the mass spectra over ten continuous scans in the corresponding time window. Three of 1518 peaks were extracted to characterize the metabolomic profile. A data matrix was constructed, with each row representing one case and each column representing one peak variable (818 rows × 1518 variables). Then, the matrix goes through normalization, natural log transform, and univariate scaling before univariate analysis, multivariate analysis, and machine learning model development. Refer to our previous report for specific metabolomics data processing [20].

### Statistical analysis

Unsupervised metabolic profile differentiation between the SP and NC groups was first conducted with t-stochastic neighbor embedding (t-SNE) in MATLAB. The rank sum test was first implemented separately between the two cohorts to search for significantly different metabolite ions between the SP and NC groups. The false discovery rate (FDR) was used as the adjusted *p*-value to assess statistical significance. An ion was selected if its FDR value was lower than 0.05. (Orthogonal) Partial least squares discriminant analysis ({O}PLS-DA} was used for SP and HC using SIMCA-P (Umetrics, Umea, Sweden). Variables with importance in projection (VIP) higher than 1.5 were considered to contribute significantly to the pattern recognition of different SP stages. Prism (GraphPad Software, United States) was used to plot box plots, heatmaps, and receiver operating characteristic (ROC) curves.

## Results

### Saliva metabolic profiling of severe periodontitis patients

**FIGURE 1:**
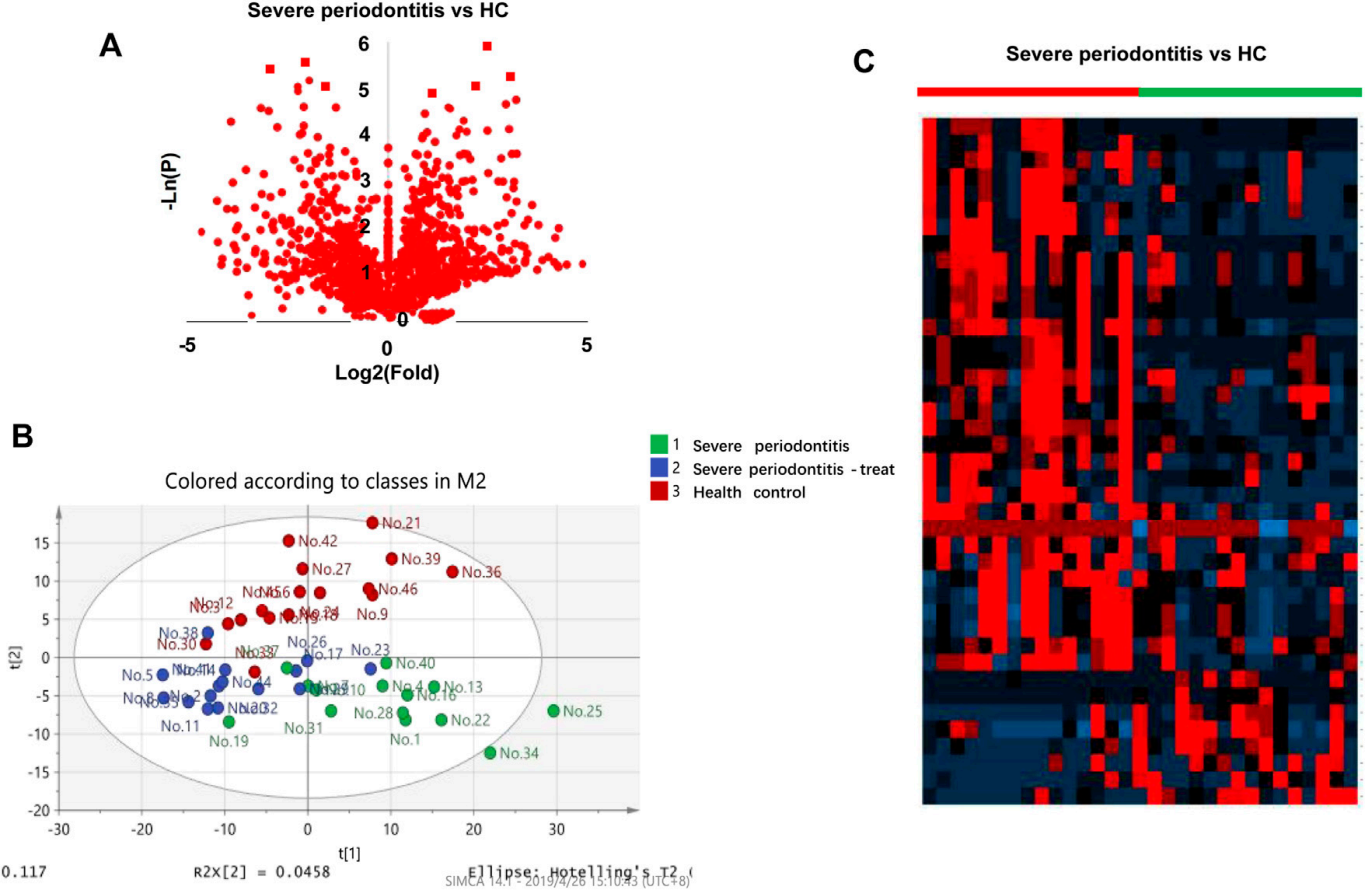
Diagram of the saliva metabolic profiling workflow by CPSI-MS/ML. **(A)** Volcano graph analysis between severe periodontitis and health control. **(B)** Saliva metabolite PLS-DA analysis in pre-treatment, post-treatment periodontitis and healthy groups. **(C)** Heatmap analysis pre-treatment severe periodontitis vs. health control.

**FIGURE 2:**
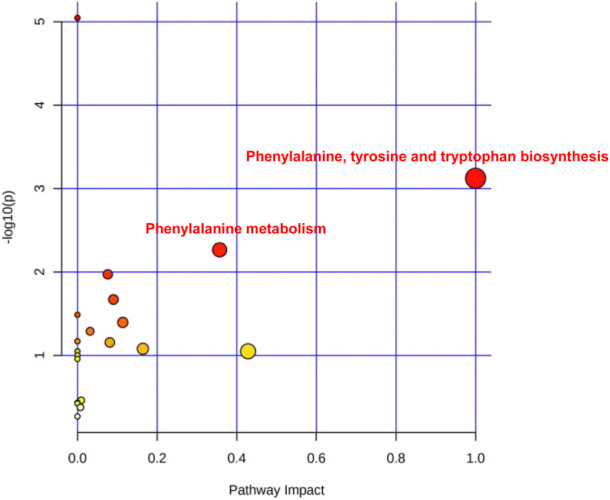
The severe periodontitis-associated metabolism pathways. Main metabolism pathways including phenylalanine, tyrosine and tryptophan biosynthesis, phenylalanine metabolism.

The collected 31 saliva cases (16 HC and 15 SP Stage IV and Grade C) were used for saliva marker discovery. A total of 301 peaks were selected to characterize the global metabolic profiles of HC and SP stages IV and C (pre- and post-treatment). The identified metabolites included amino acids, carbohydrates, lipids, and other compounds. To identify metabolic markers of SP, all metabolites were analyzed by volcano plot with fold change threshold (>1.5) and Student’s t-test threshold (*p* < 0.05) between severe periodontitis and HC (Figure 1A). Twenty metabolites were screened out (fold change >1.5, or <0.5, and *p* < 0.05), including seven amino acids (Table 1). PLS-DA and heat map analysis achieved great separation between SP and normal conditions based on the discovered metabolites (Figures 1B,C). To identify the metabolic pathways associated with the development of SP, twenty differential metabolites were performed KEGG metabolic pathway analysis (Figure 2; Table 2). The results showed that amino acid metabolism, such as tryptophan biosynthesis and phenylalanine metabolism, is closely related to the development of periodontitis.

**TABLE 1:** Differential metabolite between severe periodontitis (pre-treatment) and health control (*n* = 20).

Adduct ion	Metabolite	Theoretical	Experimental	FC	*p* value
[M + Na]+	Taurine	148.0039	148.0037	13.38045826	2.28672E-05
[M + H]+	Pipecolic acid	130.0863	130.0861	3.949052317	0.000402786
[M + K]+	Creatinine	152.0221	152.0219	0.245158934	0.003712902
[M + H]+	leucine/isoleucine	132.1019	132.1017	8.624071109	0.005064387
[M + H]+	Phenylalanine	166.0863	166.0862	4.707537574	0.006234822
[M + Na]+	Chlorate	106.9506	106.9504	0.347728868	0.006294726
[M + K]+	Glutamine	185.0323	185.0321	9.283318609	0.008748689
[M-H2O + H]+	Tyramine	120.0814	120.0806	7.788885691	0.009620617
[M + H]+	N-Acetylcadaverine	145.1335	145.1335	4.044354976	0.016704605
[M + H]+	Putrescine	89.1073	89.10713	5.632713483	0.017023921
[M + H]+	N-Acetylputrescine	131.1179	131.1178	3.343441001	0.018178897
[M + Na]+	Citric acid	215.0162	215.0163	0.213648431	0.018644655
[M + K]+	alpha-CEHC	317.115	317.1149	1.834234529	0.019062846
[M+2K-H]+	Triethanolamine	226.0242	226.0240	9.383677694	0.028211196
[M + H-H2O]+	N1-Acetylspermine	227.2236	227.2229	3.967726161	0.028620548
[M + H]+	Arginine	175.119	175.1188	1.994356976	0.028753069
[M + H]+	Lysine	147.1128	147.1127	3.485637087	0.029056012
[M + K]+	Citrulline/Argininic acid	214.0588	214.0589	7.166101262	0.031093969
[M+2K-H]+	Methylthiophene	174.9381	174.9378	0.259340288	0.040517062
[M + K]+	Tyrosine	220.0371	220.0372	2.55777336	0.047424061

**TABLE 2:** Pathway analysis of saliva metabolites of severe periodontitis.

Pathway	Total	Expected	Hits	*P*	-Log10(p)	Impact
Aminoacyl-tRNA biosynthesis	48	0.55742	6	9.00E-06	5.0459	0
Phenylalanine, tyrosine and tryptophan biosynthesis	4	0.046452	2	0.000754	3.1225	1
Phenylalanine metabolism	10	0.11613	2	0.005427	2.2654	0.35714
Arginine biosynthesis	14	0.16258	2	0.010676	1.9716	0.07614
Citrate cycle (TCA cycle)	20	0.23226	2	0.021391	1.6698	0.09038
Lysine degradation	25	0.29032	2	0.032636	1.4863	0
Alanine, aspartate and glutamate metabolism	28	0.32516	2	0.040286	1.3948	0.11378
Glyoxylate and dicarboxylate metabolism	32	0.37161	2	0.051436	1.2887	0.03175
D-Glutamine and D-glutamate metabolism	6	0.069677	1	0.067792	1.1688	0
Nitrogen metabolism	6	0.069677	1	0.067792	1.1688	0
Arginine and proline metabolism	38	0.44129	2	0.069981	1.155	0.08132
Tyrosine metabolism	42	0.48774	2	0.083408	1.0788	0.16435
Valine, leucine and isoleucine biosynthesis	8	0.092903	1	0.089408	1.0486	0
Taurine and hypotaurine metabolism	8	0.092903	1	0.089408	1.0486	0.42857
Ubiquinone and other terpenoid-quinone biosynthesis	9	0.10452	1	0.10004	0.99984	0
Biotin metabolism	10	0.11613	1	0.11055	0.95644	0
Glycerophospholipid metabolism	36	0.41806	1	0.34647	0.46034	0.00937
Pyrimidine metabolism	39	0.4529	1	0.36951	0.43237	0
Valine, leucine and isoleucine degradation	40	0.46452	1	0.37703	0.42363	0
Primary bile acid biosynthesis	46	0.53419	1	0.42035	0.37639	0.00758
Purine metabolism	65	0.75484	1	0.53952	0.268	0

### Metabolite changes after non-surgical periodontal therapy

**FIGURE 3:**
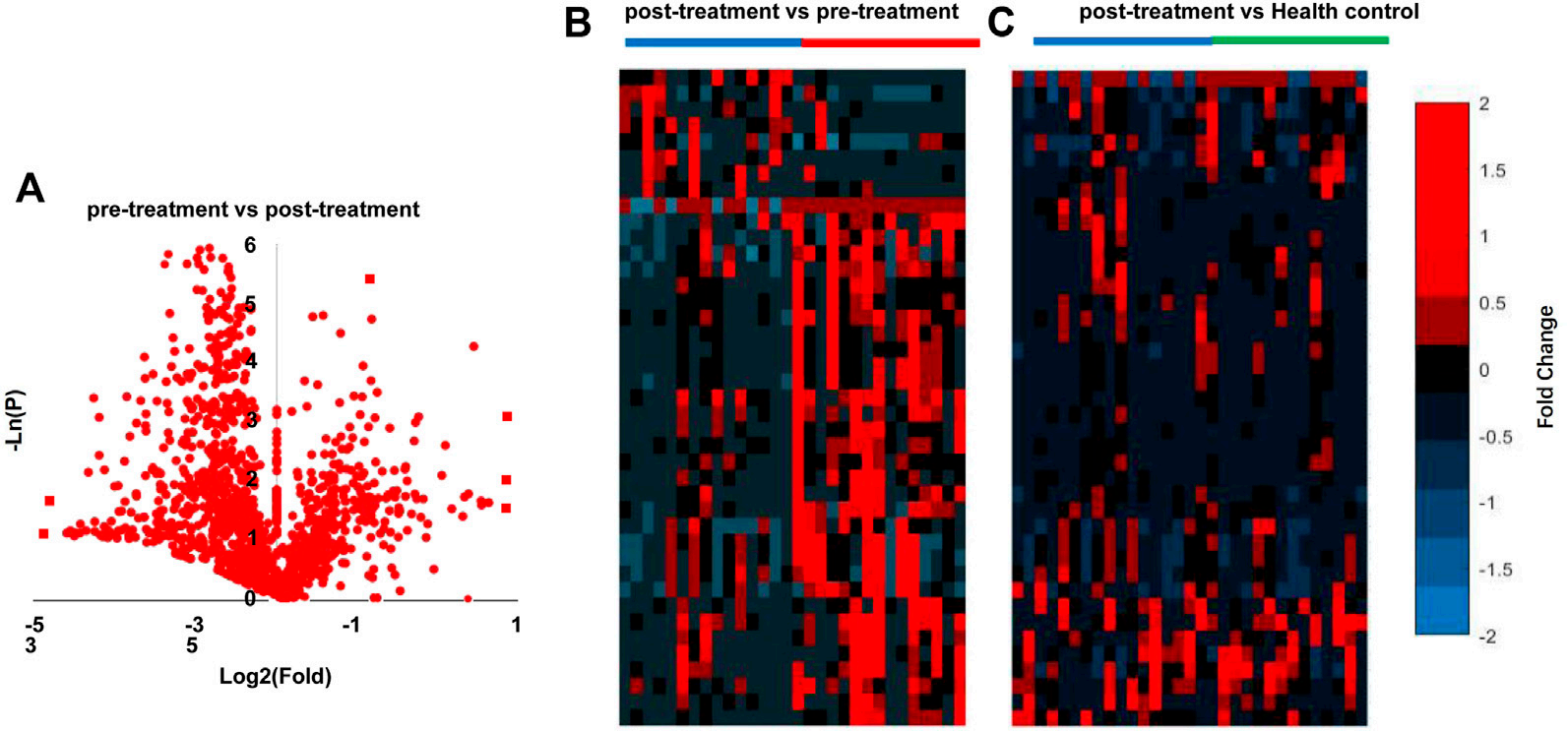
Analysis of SP pre-treatment vs. post-treatment. **(A)** Volcano graph analysis between pre-treatment and post-treatment **(B)**, heatmap analysis pre-treatment vs. post-treatment, **(C)** heatmap analysis of post-treatment vs. Health control.

**FIGURE 4:**
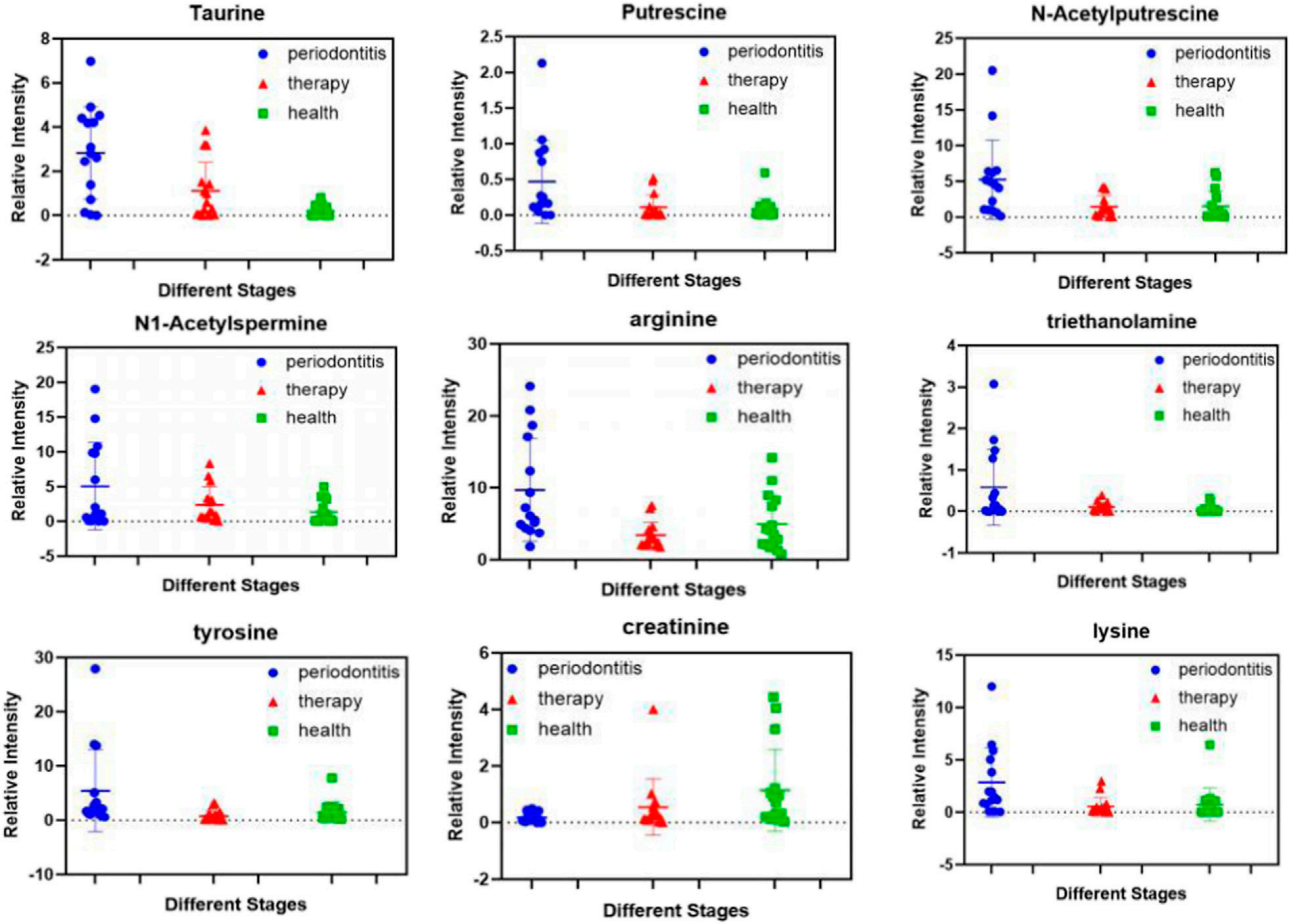
Expression levels of differential metabolites in pre-treatment, post-treatment periodontitis and healthy groups.

All 15 patients with SP (stage IV and grade C) underwent NST. Next, we evaluated whether NST could affect the metabolomic profile in the saliva of patients with SP. As shown in Table 3, twenty-four metabolites were significantly changed after NST (fold change >1.5 or <0.5 and *p* < 0.05, t-test) (Figure 3A). Heat map analysis also significantly distinguished between pre -and posttreatment (Figure 3B). However, there was no significant difference between the posttreatment periodontitis group and the healthy group (Figure 3C), which demonstrates the efficacy of NST. Figure 4 shows the relative intensities of the common metabolites in the three groups. These results indicated that salivary metabolites in the oral cavity were significantly altered after plaque removal.

**TABLE 3:** Differential metabolite between post-treat and pre-treat group (*n* = 31).

Adduct ion	Metabolite	Theoretical (m/z)	Experimental (m/z)	FC	*p* value
[M + CH3OH + H]+	1,3-Dimethyluracil	173.0921	173.0919	0.115106013	0.033375369
[M + H]+	Glutamic acid	148.0604	148.0603	0.148412844	0.017026785
[M + K]+	Citrulline/Argininic acid	214.0588	214.0589	0.151950348	0.044749385
[M-H2O + H]+	tyramine	120.0814	120.0806	0.167856224	0.022762496
[M + Na]+	Amaranth	560.9703	560.9729	0.178158531	0.036095169
[M + K]+	N-Acetyl-glucosamine or Glucosamine 6-phosphate	260.0531	260.0533	0.206227911	0.037398582
[M + H]+	Imidazolepropionic acid or 1,3-Dimethyluracil	141.0659	141.0657	0.211537893	0.043361772
[M + H]+	Lysine	147.1128	147.1127	0.219735916	0.019980917
[M + H]+	Phenylalanine	166.0863	166.0862	0.224486539	0.012273488
[M + H]+	Putrescine	89.1073	89.10713	0.229320503	0.015428116
[M + K]+	Glutamine	185.0323	185.0321	0.237444141	0.024112654
[M + H]+	leucine/isoleucine	132.1019	132.1017	0.253025803	0.025737485
[M + H]+	N-Acetylputrescine	131.1179	131.1178	0.284009334	0.014723675
[M + H]+	Ornithine	133.0972	133.0970	0.288444073	0.03882212
[M + K]+	Homocitrulline	228.0745	228.0744	0.318278237	0.034673258
[M + K]+	Tyrosine	220.0371	220.0372	0.326325537	0.005348209
[M + H]+	Pipecolic acid	130.0863	130.0861	0.337642644	0.002353984
[M + H]+	Arginine	175.119	175.1188	0.341621155	0.002713833
[M + Na]+	Adenosine monophosphate	370.0523	370.0513	0.34623741	0.038844112
[M + H]+	N-Acetylcadaverine	145.1335	145.1335	0.380520717	0.00782618
[M + Na]+	3-Oxoalanine	126.0162	126.0170	0.393187433	0.043574971
[M + Na]+	taurine	148.0039	148.0037	0.410119021	0.00823438
[M + K]+	Phosphocreatine	249.999	250.0032	1.802868539	0.027251416
[M+2K-H]+	Isoniazid alpha-ketoglutaric acid	341.9889	341.9881	3.041355093	0.033010147

## Discussion

The aim of this pilot study was to identify metabolites that differentiate severe periodontitis Stage IV and Grade C from healthy people and to reflect the changes in metabolites in patients with SP after NST using CPSI-MS.

CPSI-MS is an advanced ambient ionization MS technique, which is used for identifying the covalent structure of a metabolite and probing the variations that occur in human diseases. CPSI has become a very powerful tool for characterizing large biomolecular systems, with strong separation ability, high sensitivity, large peak capacity, excellent repeatability, and stable retention time. The key advantage of the above-described CPSI-MS procedure is that it is high-throughput and therefore well suited for big cohort analysis. Compared to conventional mass spectrometry, CPSI has no additional purification or enrichment, the sample directly loaded to form a dried biofluid spot. Both desorption and ionization can be triggered by applying an extraction solvent and a high voltage, which is as simple as operating an on/off switch. Thus, CPSI-MS is suitable for rapid, direct metabolic profiling of complex biological fluids such as serum, plasma, saliva, urine, and whole blood [20]. Moreover, direct metabolic profiling of a single case takes only a few seconds, which can collect sufficient MS data. The total analytical period for these saliva samples collected from volunteers took 0.5 h, which means that CPSI-MS can analyze approximately 100 samples within 1.2 h. In addition, the conductive polymer composite (MWCNT/PMMA) used in CPSI-MS is inexpensive, clean, and consumable, which facilitates the economic testing of large-scale samples [20].

Saliva contains a complex and variable mixture of metabolites of heterogeneous origin (e.g., host, diet, medication, and microbes) [21]. In this untargeted metabolomics study, it was possible to separate patients with SP from HCs using CPSI-MS. The present study successfully screened 20 differential metabolites in saliva samples as biomarkers to distinguish between patients with SP and HCs. The levels of glutamate, glutamine, leucine, and isoleucine in patients with SP were significantly higher than those in the HCs. Metabolomics studies of periodontitis, particularly stage IV grade C periodontitis, are limited, and our study provides potentially predictable or diagnostic metabolic markers. Moreover, KEGG metabolic pathway analysis indicated that SP may be related to the biosynthesis of aminoacyl-tRNA and arginine, lysine degradation, and amino acid metabolism (phenylalanine, alanine, aspartate, glutamate, and tyrosine). All these results suggest that there may be active amino acid metabolism in the saliva of patients with SP, and our results are consistent with a previous report [21–24].

Single metabolite fluctuations could be linked to different events occurring in health controls and disease metabolite producing changes after treatment. All 15 patients with SP received NST. Interestingly, the present study detected 24 differential metabolites after NST, and the concentration of most metabolites decreased after NST treatment, whereas the concentrations of phosphocreatine and alpha-ketoglutaric acid increased (*p* < 0.05). In this context, we observed that the levels of tyrosine and isoleucine decreased after NST (*p* < 0.005), as they reflect the host immune response to the oral microbiome and have been associated with active periodontal disease. The decreased phenylalanine level could be a consequence of reduced host tissue degradation after NST.

To the best of our knowledge, many differential metabolite variables between patients with SP and HCs have yet to be identified. The task is challenging because metabolomicss have a large and unknown complexity, and their identification may have been limited by their intrinsic properties and the single metabolomics technology platform used in this study. It can therefore be anticipated that larger sample sizes and a combination of multiple analytical platforms are required to validate the results of the present study.

## Data Availability

The original contributions presented in the study are included in the article/supplementary material, further inquiries can be directed to the corresponding authors.

## Abbreviations

CPSI-MS, Conductive polymer spray ionization mass spectrometry; CPSI-MS/ML, Conductive polymer spray ionization mass spectrometry combine with machine learning; FDR, false discovery rate; GC-MS, Gas chromatography mass spectrometry; HC, healthy control; LC-MS, liquid chromatography mass spectrometry; NMR, nuclear magnetic resonance spectrometry; NST, nonsurgical periodontal therapy; (O)PLS-DA, (Orthogonal) Partial least squares discriminant analysis; OSCC, oral squamous cell carcinoma; PML, premalignant lesion; QC, Quality control; ROC, receiver operating characteristic; SP, severe periodontitis; TIC, total ion current; t-SNE, t-stochastic neighbor embedding; VIP, Variables with importance in projection.
